# Efficient bioproduction of 5-aminolevulinic acid, a promising biostimulant and nutrient, from renewable bioresources by engineered *Corynebacterium glutamicum*

**DOI:** 10.1186/s13068-020-01685-0

**Published:** 2020-03-10

**Authors:** Jiuzhou Chen, Yu Wang, Xuan Guo, Deming Rao, Wenjuan Zhou, Ping Zheng, Jibin Sun, Yanhe Ma

**Affiliations:** Key Laboratory of Systems Microbial Biotechnology, Tianjin Institute of Industrial Biotechnology, Chinese Academy of Sciences, Tianjin, 300308 China

**Keywords:** 5-Aminolevulinic acid, Renewable bioresource, Cassava bagasse, *Corynebacterium glutamicum*, Metabolic engineering

## Abstract

**Background:**

5-Aminolevulinic acid (5-ALA) is a promising biostimulant, feed nutrient, and photodynamic drug with wide applications in modern agriculture and therapy. Considering the complexity and low yield of chemical synthesis methods, bioproduction of 5-ALA has drawn intensive attention recently. However, the present bioproduction processes use refined glucose as the main carbon source and the production level still needs further enhancement.

**Results:**

To lay a solid technological foundation for large-scale commercialized bioproduction of 5-ALA, an industrial workhorse *Corynebacterium glutamicum* was metabolically engineered for high-level 5-ALA biosynthesis from cheap renewable bioresources. After evaluation of 5-ALA synthetases from different sources, the 5-ALA biosynthetic pathway and anaplerotic pathway were rebalanced by regulating intracellular activities of 5-ALA synthetase and phosphoenolpyruvate carboxylase. The engineered biocatalyst produced 5.5 g/L 5-ALA in shake flasks and 16.3 g/L in 5-L bioreactors with a one-step fermentation process from glucose. To lower the cost of feedstock, cheap raw materials were used to replace glucose. Enzymatically hydrolyzed cassava bagasse was proven to be a perfect alternative to refined sugars since the final 5-ALA titer further increased to 18.5 g/L. Use of corn starch hydrolysate resulted in a similar 5-ALA production level (16.0 g/L) with glucose, whereas use of beet molasses caused seriously inhibition. The results obtained here represent a new record of 5-ALA bioproduction. It is estimated that replacing glucose with cassava bagasse will reduce the carbon source cost by 90.1%.

**Conclusions:**

The high-level biosynthesis of 5-ALA from cheap bioresources will brighten the prospects for industrialization of this sustainable and environment-friendly process. The strategy for balancing metabolic flux developed in this study can also be used for improving the bioproduction of other value-added chemicals.

## Background

5-Aminolevulinic acid (5-ALA), a non-proteingenic amino acid that widely exists in microbe, plant and animal cells, is a common precursor of tetrapyrrole compounds, such as heme, porphyrin, chlorophyll, and vitamin B_12_. Because of the critical roles of tetrapyrrole compounds in regulating cell metabolism and growth, 5-ALA has gained increasing attention in the fields of medicine, agriculture and livestock [1, 2]. As a new generation of photosensitizer, 5-ALA has displayed wide applications in photodynamic diagnosis and therapy for various cancers and many skin diseases [3]. In agriculture, exogenous supplement with 5-ALA could effectively increase intracellular chlorophyll or heme levels in crop, livestock, and poultry. Therefore, 5-ALA can be used as a plant growth regulator to promote the yield and resist the harmful effects caused by various abiotic stresses [4]. 5-ALA can also be used as feed additives to enhance ATP production and immune response, leading to improved growth performance of chicken, pig, white shrimp, etc. [5–7]. Therefore, 5-ALA has been considered as a promising biostimulant for crop production and a feed nutrient for livestock breeding. It is estimated that the global market size of 5-ALA will reach US$ 110 million by 2024, from US$ 86 million in 2019 [8].

Chemical synthesis is the primary method for industrial production of 5-ALA. Six chemically synthetic methods have been reported, whereas the complicated reaction and purification steps and low yield cause the high price of 5-ALA, which seriously limits its widespread use in agriculture [1]. Bioproduction of 5-ALA holds the potential to simplify the production process and lower the cost, and thus has received growing attention recently [9, 10]. Although some algae and photosynthetic bacteria are capable to synthesize 5-ALA naturally, the production levels are not satisfactory and these microorganisms are usually difficult to engineer [11, 12]. With the development of metabolic engineering and synthetic biology, intensive studies have concentrated on engineering platform microorganisms such as *Escherichia coli* and *Corynebacterium glutamicum* for 5-ALA bioproduction. A native C5 pathway that converts glutamate to 5-ALA via three enzymatic reactions exists in both *E. coli* and *C. glutamicum* [13]. By strengthening this biosynthetic pathway, 5-ALA production was achieved [14–20], but the highest titer and productivity were only 5.25 g/L and 0.16 g/L h, respectively [20]. To improve the 5-ALA production level, the exogenous C4 pathway for 5-ALA biosynthesis originated from photosynthetic bacteria was introduced into *E. coli* and *C. glutamicum* by expressing the 5-ALA synthetase (ALAS) catalyzing the condensation of succinyl-CoA and glycine to 5-ALA. Several strategies have been applied to further enforce the C4 biosynthetic route, such as enzyme screening [21–26], pathway engineering [27–31], tolerance engineering [32], and fermentation process optimization [27, 33]. By reinforcing the native antioxidant defense system in an ALAS‐expressing *E. coli* strain to combat with the reactive oxygen species generated by 5-ALA, Zhu et al. obtained the highest 5-ALA titer (11.5 g/L) of one‐step fermentation [32]. Yang et al. constructed a 5-ALA producing *C. glutamicum* by expressing a codon-optimized ALAS from *Rhodobacter capsulatus* and deactivating the succinyl‐CoA synthetase. By separating the growth and production phases, the engineered strain produced 14.7 g/L 5-ALA [27]. However, the two-step fermentation strategy consisting of cultivating, collecting, and resuspending cells in a new buffer may be challenging for large‐scale production.

So far, all the reported 5-ALA bioproduction processes rely on using glucose as the main carbon source (Table 1). Based on an economic analysis of a 10,000 tons pilot scale 5-ALA bioproduction process, we estimate that glucose cost accounts for approximately 12.5% of the total cost. To popularize application of 5-ALA in agriculture, further cost reduction is required. Therefore, cheap raw materials, such as molasses, cassava bagasse and woody biomass, are preferred to replace refined sugars. Although such cheap bioresources have been used for the bioproduction of several chemicals and biofuels [34–36], they have not been explored for 5-ALA production so far. Moreover, improving the conversion yield of the carbon source to 5-ALA and the final titer by metabolic engineering is also favorable for reducing the production cost of 5-ALA.

**Table 1:** Bioproduction of 5-ALA by engineered strains via C4 biosynthetic pathway from different substrates

Strain and strategy	Main substrates	Titer (g/L)	Productivity (g/L h)	Reference
*E. coli*				
Overexpression of hemA from *R. sphaeroides*	Succinate, glycine	5.2	0.43	[21]
Overexpression of *hemA* from *R. palustris* KUGB306	Glucose, succinate, glycine	5.2	0.32	[23]
Overexpression of *hemA* from *R. sphaeroides* 2.4.1	Glucose, succinate, glycine	6.6	0.24	[22]
Overexpression of *hemA* from *Agrobacterium radiobacter* zju-0121	Glucose, succinate, glycine, xylose	7.3	0.24	[24]
Overexpression of *hemO* from *R. palustris* ATCC 17001	Glucose, succinate, glycine	6.3	0.26	[25]
Overexpression of *hemA* from *A. radiobacter* zju-0121, short-term dissolved oxygen shock	Glucose, succinate, glycine	9.4	0.43	[33]
Overexpression of *hemA* from *R. capsulatus*	Glycerol, succinate, glycine	8.8	0.24	[26]
Overexpression of *hemA* from *R. capsulatus*, pathway optimization for CoA and precursor biosynthesis, downregulation of *hemB*	Glucose	2.8	0.06	[29]
*C. glutamicum*				
Overexpression of *hemA* from *R. sphaeroides*, native *ppc* and *rhtA* from *E. coli*, deletion of *ldhA*, *pqo*, *cat*, *pta*, *ackA* and *pbp1b*	Glucose, glycine	7.5	0.23	[28]
Overexpression of *hemA* from *R. capsulatus* SB1003, deletion of *sucCD*	Glucose, glycine	7.6	0.095	[27]
Overexpression of *hemA* from *R. capsulatus* SB1003 and *rhtA* from *E. coli*, deletion of *sucCD*	Glucose, glycine	14.7^a^	0.92^a^	
Moderate overexpression of *hemA* from *R. palustris* ATCC 17,001 and native *ppc* to balance 5-ALA biosynthetic and anaplerotic pathways	Glucose, glycine	16.3	0.42	This study
	Cassava bagasse hydrolysate, glycine	18.5	0.47	

^a^A two-stage fermentation was employed. The cultivation time of the first step for cell growth was not reported in that study, and thus the productivity of the second step for 5-ALA production was shown

**Fig. 1:**
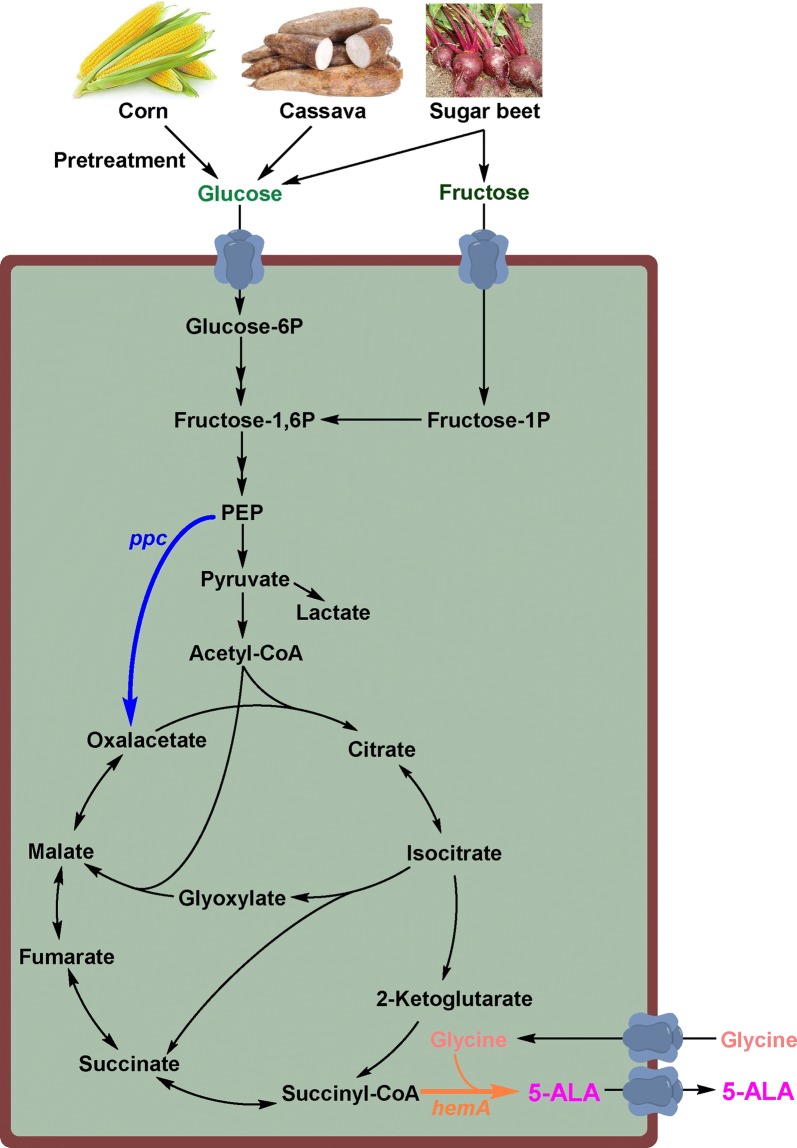
Schematic illustration of the bioproduction of 5-ALA by metabolically engineered *C. glutamicum* from cheap renewable bioresources. PEP, phosphoenolpyruvate; *hemA*, 5-aminolevulinate synthase (ALAS) encoding gene; *ppc*, phosphoenolpyruvate carboxylase (PPC) encoding gene

In this work, a superior 5-ALA producer was constructed based on *C. glutamicum* by screening ALAS candidates and balancing precursor supply and 5-ALA biosynthesis via regulating intracellular enzyme activities (Fig. 1). By this means, 16.3 g/L 5-ALA was produced from glucose and glycine using a one-step fermentation. To further decrease the cost of feedstock, different cheap carbon sources, including cassava bagasse, corn starch, and beet molasses, were used to replace glucose. The highest 5-ALA titer (18.5 g/L) was obtained using enzymatically hydrolyzed cassava bagasse. The metabolic balance strategy developed here can also be applied for improving the bioproduction of other value-added chemicals. The high-level biosynthesis of 5-ALA from cheap bioresources represents a crucial step in industrialization of 5-ALA bioproduction.

## Results and discussion

### ALAS screening for 5-ALA production in *C. glutamicum*

**Fig. 2:**
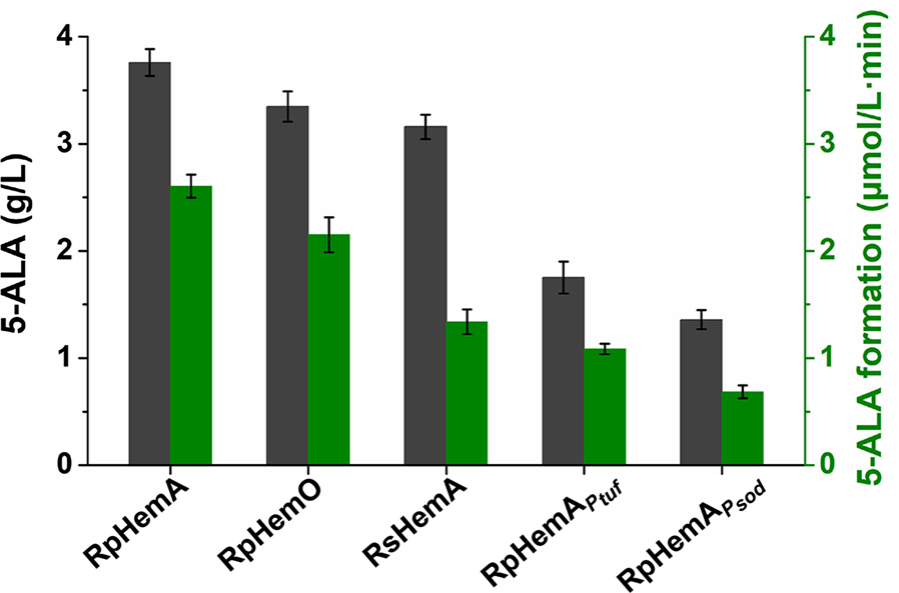
Screening of ALASs from different sources for 5-ALA production. RpHemA, *hemA* from *R. palustris*, expressed using *P*_*trc*_; RpHemO, *hemO* from *R. palustris*, expressed using *P*_*trc*_; RsHemA, *hemA* from *R. sphaeroides*, expressed using *P*_*trc*_; RpHemA_*Ptuf*_, *hemA* from *R. palustris*, expressed using *P*_*tuf*_; RpHemA_*Psod*_, *hemA* from *R. palustris*, expressed using *P*_*sod*_. Dark grey bars, 5-ALA production level; green bars, ALAS activity in crude extract. Strains with different ALASs were cultivated in shake flasks with modified M9 medium. The titer of 5-ALA at 36 h is shown. Cells were collected at the same time and lysed for ALAS activity assay. The reaction mixture contained 100 mM Tris–HCl (pH 7.5), 200 mM glycine, 0.2 mM succinyl-CoA, 0.1 mM pyridoxal phosphate (PLP) and 20 µg crude extract. After proceeding at 37 ℃ for 10 min, the reaction was terminated by the addition of 10% (v/v) trichloroacetic acid. Concentration of 5-ALA in the supernatant was determined. Error bars indicate standard deviations from three parallel experiments

Since ALAS is an essential enzyme for 5-ALA production via C4 pathway and it is absent from *C. glutamicum*, introduction of a heterogeneous ALAS is required (Fig. 1). We previously identified two ALASs with superior activities, RpHemA and RpHemO, from *Rhodopseudomonas palustris* ATCC 17001 [25]. They were expressed via plasmid under the control of isopropyl-β-d-thiogalactopyranoside (IPTG)-inducible promoter *P*_*trc*_ and evaluated for 5-ALA production in *C. glutamicum*, together with another reported ALAS from *Rhodobacter sphaeroides* (RsHemA). *C. glutamicum* strain CA expressing RpHemA showed the highest ALAS activity in crude extract (2.6 µmol/L min), followed by strains expressing RpHemO (2.2 µmol/L min) and RsHemA (1.3 µmol/L min) (Fig. 2). The 5-ALA titers were in line with the trend of ALAS activity. Compared with the other two ALASs, RpHemA was the best candidate for 5-ALA production in *C. glutamicum*, and the 5-ALA titer reached to 3.8 g/L, which was 15.2% and 18.9% higher than those produced by strains expressing RpHemO and RsHemA, respectively. These data indicated that 5-ALA production was closely related to the intracellular ALAS activity.

### Optimizing ALAS expression for enhanced 5-ALA production via ribosome binding site (RBS) engineering

**Fig. 3:**
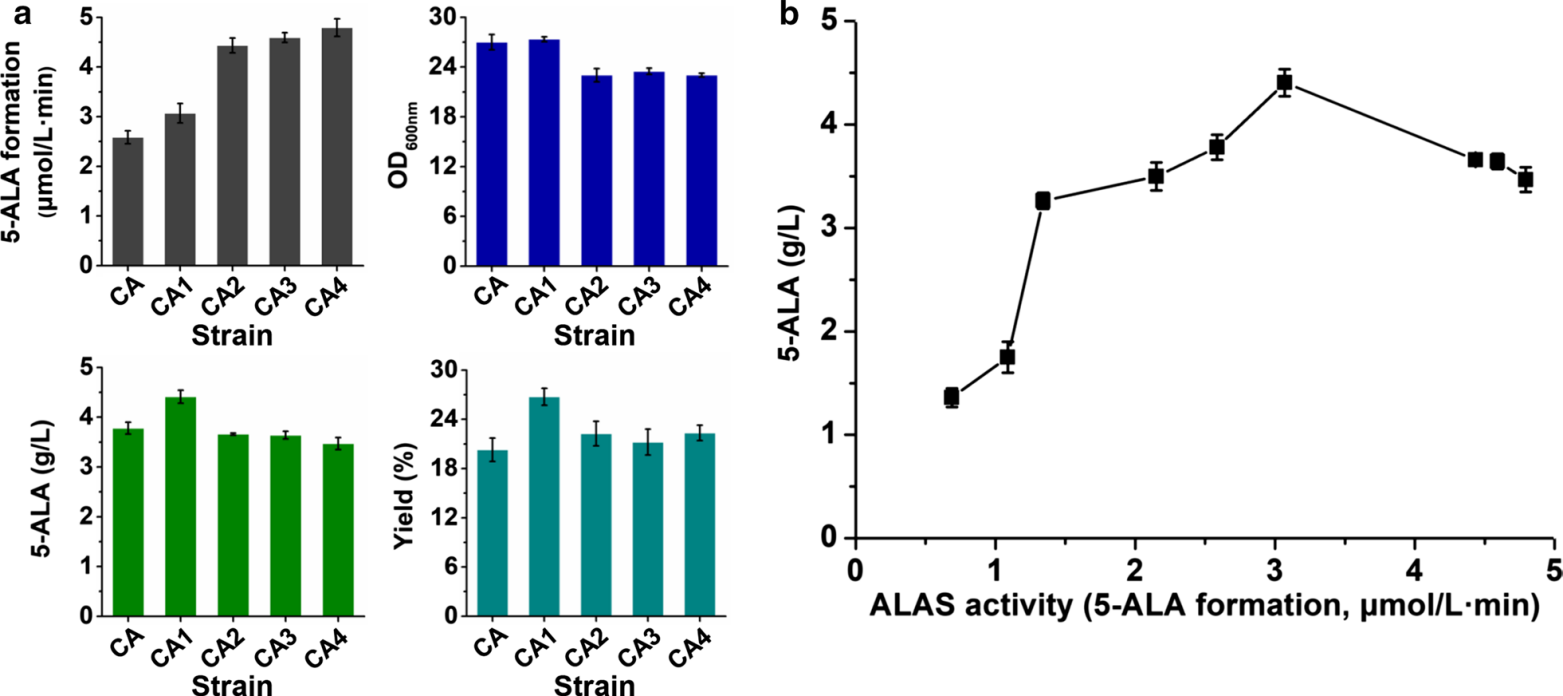
Fine-tuning of ALAS expression for 5-ALA production. **a** Effects of regulating ALAS expression via RBS engineering on 5-ALA production. **b** Correlation between ALAS activity in crude extract and 5-ALA production level. The *x*-axis shows mean of ALAS activities in crude extract measured by ALAS formation rate of three parallel experiments. Strains were cultivated in shake flasks with modified M9 medium. OD_600nm_ value, 5-ALA titer and yield at 36 h are shown. Cells were collected at the same time and lysed for ALAS activity assay. The reaction mixture contained 100 mM Tris–HCl (pH 7.5), 200 mM glycine, 0.2 mM succinyl-CoA, 0.1 mM pyridoxal phosphate (PLP) and 20 µg crude extract. After proceeding at 37 ℃ for 10 min, the reaction was terminated by the addition of 10% (v/v) trichloroacetic acid. Concentration of 5-ALA in the supernatant was determined. Error bars indicate standard deviations from three parallel experiments

It is speculated that optimizing intracellular ALAS activity can further improve 5-ALA biosynthesis. Firstly, two native strong constitutive promoters *P*_*tuf*_ and *P*_*sod*_ [37] were first used to replace the IPTG-inducible one to regulate transcription of ALAS encoding gene. However, promoter replacement led to decreased intracellular ALAS activity and consequently decreased 5-ALA titer (Fig. 2), which were consistent with the fact that these two constitutive promoters exhibit lower activities relative to IPTG-inducible ones [37]. We then applied RBS engineering to fine-tune the translation level of ALAS and increase intracellular ALAS activity. Four RBSs (RBS-2, AAAGGAGGTTGTC; RBS-3, AAAGGAGCGGTCC; RBS-4, AAAGGAGGATTAG; RBS-5, AAAGGAGTTGCTT) with relatively high translational activities were selected from previously constructed libraries [38] to replace the original RBS of ALAS encoding gene in strain CA (RBS-1, AAGGAGATATAGAT) and produced four recombinant strains designated as CA1, CA2, CA3, and CA4. All the four engineered strains exhibited increased ALAS activity in crude extract, and the highest activity reached 4.8 µmol/L min in strain CA4, nearly twice as much as that of strain CA (Fig. 3a). With the increase of intracellular ALAS activity, the 5-ALA production level ascended first and then descended. The highest 5-ALA titer (4.4 g/L, 15.8% improvement) and molar yield (26.8%, 32.0% improvement) were achieved by strain CA1, of which the corresponding ALAS activity increased by 11% compared to the parent strain CA. When the intracellular ALAS activity further increased, growth inhibition was observed, accompanied by a decrease in 5-ALA production (Fig. 3a).

Based on the data obtained here, the correlation between ALAS activity and 5-ALA production was concluded (Fig. 3b). Although ALAS is a prerequisite for 5-ALA biosynthesis, its expression level is not as high as possible for the product formation. The ALAS activity in crude extract around 3.1 µmol/L min mostly favored 5-ALA production in *C. glutamicum*. The decreases in 5-ALA production level and biomass were possibly caused by the unbalanced 5-ALA biosynthetic pathway and TCA cycle. Succinyl-CoA, the precursor of 5-ALA biosynthesis, is also an intermediate of TCA cycle and plays an important role in a variety of metabolic processes [39]. The over-consumption of succinyl-CoA by excessive ALAS enzyme may slow down TCA cycle and affect energy metabolism, resulting in a tradeoff effect like growth inhibition observed here (Fig. 3a). Furthermore, production of large amounts of ALAS protein would require substantial resources, which would lay a heavy metabolic burden on cell maintenance and propagation. Thus, moderate expression of ALAS was optimal for maximizing 5-ALA production in *C. glutamicum*.

### Balancing 5-ALA biosynthetic pathway and anaplerotic pathway for enhanced 5-ALA production

**Fig. 4:**
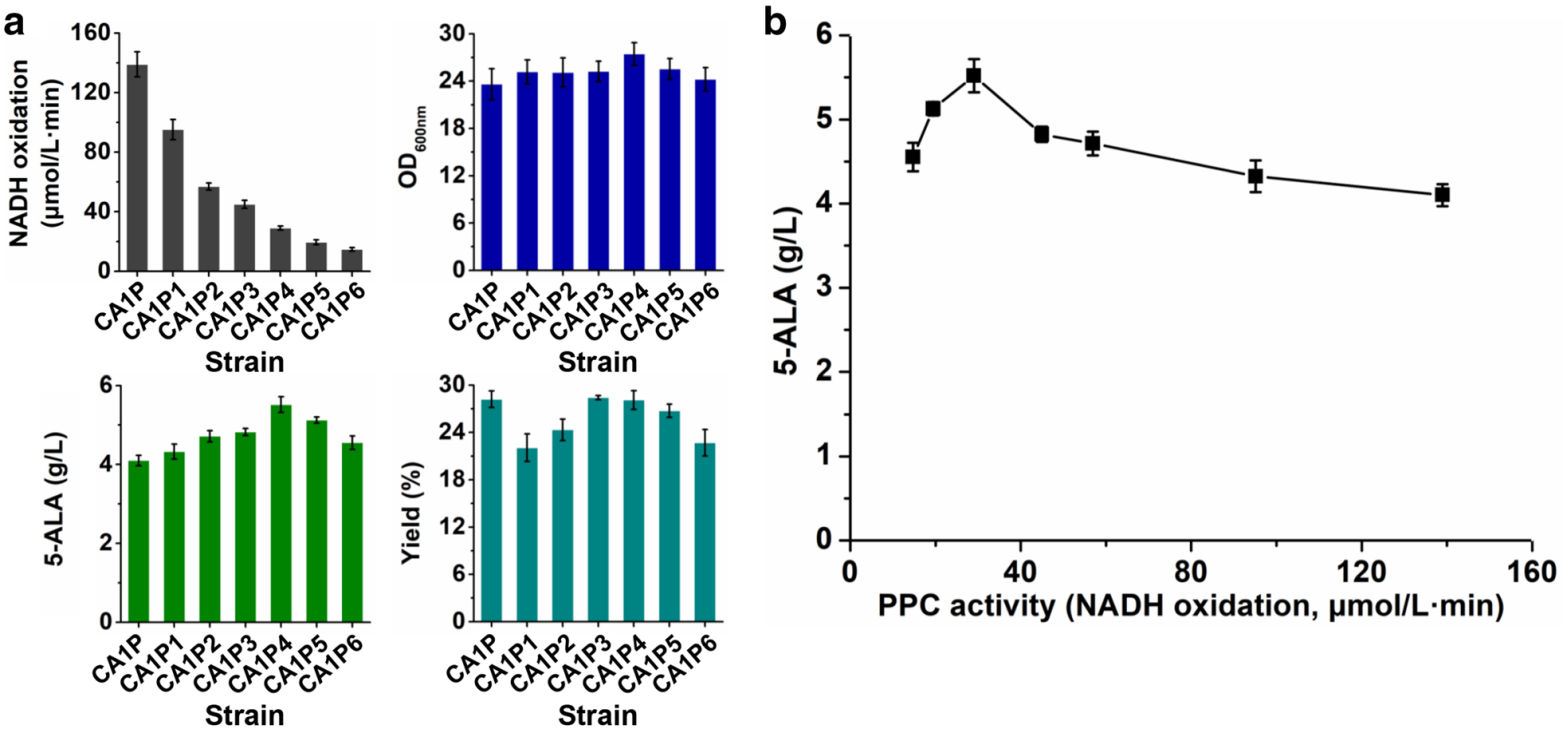
Fine-tuning of PPC expression for 5-ALA production. **a** Effects of regulating PPC expression via RBS engineering on 5-ALA production. **b** Correlation between PPC activity in crude extract and 5-ALA production level. The *x*-axis shows mean of PPC activities in crude extract measured by NADH oxidation rate of three parallel experiments. Strains were cultivated in shake flasks with modified M9 medium. OD_600nm_ value, 5-ALA titer and yield at 36 h are shown. Cells were collected at the same time and lysed for PPC activity assay. The PPC activity was determined by a coupling reaction catalyzed by malate dehydrogenase at 30 ℃. The reaction mixture contained 100 mM Tris–HCl (pH 7.5), 2 mM phosphoenolpyruvate, 10 mM NaHCO_3_, 10 mM MnSO_4_, 0.1 mM NADH, 20% (v/v) glycerol, 1.6 U malate dehydrogenase, and 20 µg crude extract. The activity was assayed spectrophotometrically by monitoring the decrease in absorbance of NADH at 340 nm. Error bars indicate standard deviations from three parallel experiments

Considering 5-ALA biosynthesis withdraws carbon flux from TCA cycle, enhancement of the anaplerotic pathway for oxaloacetate should favor 5-ALA production. Overexpression of phosphoenolpyruvate carboxylase (PPC) has been proven to be effective to improve biosynthesis of oxaloacetate derivatives such as l-lysine, l-glutamate, and succinate [40–42]. Therefore, the native PPC of *C. glutamicum* was overexpressed under the control of the same *P*_*trc*_ promoter with ALAS in *C. glutamicum* strain CA1. The resultant strain CA1P exhibited a 46.3-fold increase in intracellular PPC activity, demonstrating the successful overexpression. However, a slight decrease in 5-ALA titer was observed, which was possibly caused by the 26.3% decrease in intracellular ALAS activity (Figs. 3a, 4a). It has been reported that the expression levels of two proteins in a co-expression system are relatively lower than that of an individually expressed protein [43]. The results suggest that the excessive expression of PPC not only is unmatched with the cellular metabolism, but also competes with ALAS expression for resources.

To balance the 5-ALA biosynthetic pathway and anaplerotic pathway, PPC expression was optimized in strain CA1P using six RBSs with different translational activities (RBS-2, AAAGGAGGTTGTC; RBS-5, AAAGGAGTTGCTT; RBS-6, AAAGGAATTGGC; RBS-7, AAAGGTTTCAAGT; RBS-8, AAAGGCTGGAATT; RBS-9, AAAGGGTATTGGC) selected from reported RBS libraries [38]. Gradually decreased intracellular PPC activities were observed in engineered strains (Fig. 4a). Similar with the effect of regulating ALAS expression, 5-ALA production was first increased and then decreased along with the decrease in intracellular PPC activity. When the PPC activity in crude extract was 29.0 µmol/L min, the highest 5-ALA titer (5.5 g/L) was obtained by strain CA1P4 (Additional file 1: Fig. S1), which was increased by 34.1% compared with that of strain CA1P without RBS optimization. Cell growth was also recovered due to the moderate expression of PPC (Fig. 4a). Based on the data obtained here, the correlation between intracellular PPC activity and 5-ALA production was also concluded (Fig. 4b). Obviously, a proper reinforcement of PPC activity is favorable for 5-ALA. The intracellular ALAS activity of strain CA1P4 was 2.9 µmol/L min, which was very close to that of strain CA1 (3.1 µmol/L min) (Figs. 3a, 4a). This also suggests the balance between 5-ALA biosynthetic pathway and anaplerotic pathway is crucial for efficient 5-ALA production.

The strategy of strengthening the anaplerotic pathway for oxaloacetate has been applied for 5-ALA biosynthesis, but only limited improvement was achieved. Feng et al. replaced the native promoter of PPC with a strong promoter in a recombinant *C. glutamicum* strain expressing ALAS and this modification increased 5-ALA production by approximately 7.3%. This encouraged the authors to further strengthen the anaplerotic pathway by overexpressing pyruvate carboxylase or deleting phosphoenolpyruvate carboxykinase, but these strategies failed to continuously enhance 5-ALA production [28]. In both the literatures and our cases, simply reinforcing the anaplerotic pathway has no positive and even negative effect on 5-ALA production. A finer tuning of expression of key enzymes is strongly suggested to balance complicated pathways. Since biosynthesis of many target products derived from TCA cycle, such as succinic acid and γ-aminobutyric acid, can be potentially enhanced via strengthening the anaplerotic pathway for oxaloacetate [44, 45], the strategy presented here should be also useful for optimization of other bioproduction processes.

### Fed-batch fermentation for 5-ALA production from glucose in 5-L bioreactors by *C. glutamicum* CA1P4

**Fig. 5:**
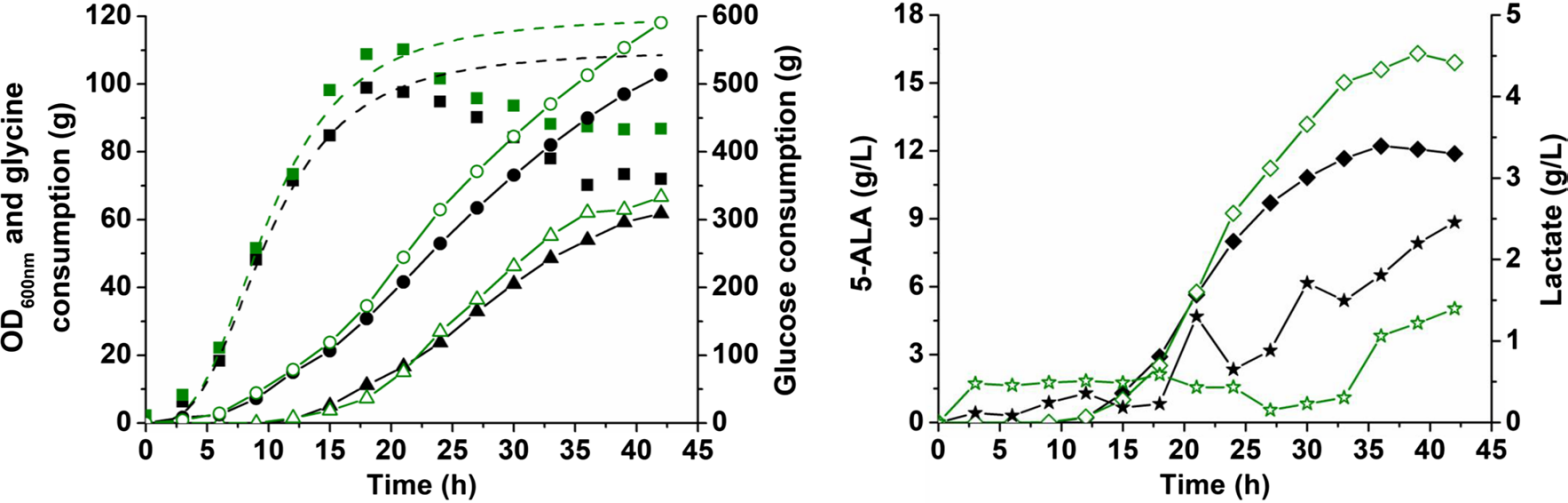
Fed-batch fermentation for 5-ALA production from glucose using strains CA and CA1P4. Black filled labels represent strain CA and green open labels represent strain CA1P4. Square, OD_600nm_; circle, glucose consumption; triangle, glycine consumption; diamond, 5-ALA; star, lactate. Dotted line represents simulated growth curve based on OD_600nm_ data. Cultivation was performed in 5-L bioreactors with fermentation medium. IPTG (0.1 mM) and glycine (4 g/L) were added when OD_600nm_ reached approximately 40 to induce gene expression and 5-ALA biosynthesis. Glucose and glycine were continuously fed into the bioreactor during the fermentation. The pH was controlled at 6.5 initially and switched to 6.0 at 15 h. The dissolved O_2_ was maintained at 30% initially and switched to 10% at 18 h

To test the high-level 5-ALA production potential of engineered strains, fed-batch fermentations were conducted in 5-L bioreactors using the optimized producer strain CA1P4 and the primary producer strain CA as a control. Overall, strain CA1P4 dramatically outmatched strain CA regarding cell growth, substrate uptake, 5-ALA production, and by-product formation (Fig. 5). Strain CA entered the stationary phage at 18 h with an OD_600nm_ peak value of 98.9, while strain CA1P4 had a prolonged exponential phage and increased the highest OD_600nm_ by 11.4% to a value of 110.2 with 15.1% more glucose consumption. After 39 h fermentation, 16.3 g/L of 5-ALA was produced by strain CA1P4, representing a 33.6% improvement compared to strain CA (12.2 g/L) (Fig. 5). Lactate was the main by-product of this fermentation process and its formation also decreased from 2.5 g/L for strain CA to 1.4 g/L for strain CA1P4, without disruption of the lactate-forming pathway (Fig. 5). The results suggest that enhancing oxaloacetate replenishment by moderate PPC overexpression avoids excess accumulation of phosphoenolpyruvate and channels more carbon flux to 5-ALA biosynthesis. All these results suggested that optimizing the metabolic carbon flux by precise control of key enzyme activities is a highly effective strategy to improve 5-ALA production.

### Efficient 5-ALA production from cheap renewable bioresources

**Fig. 6:**
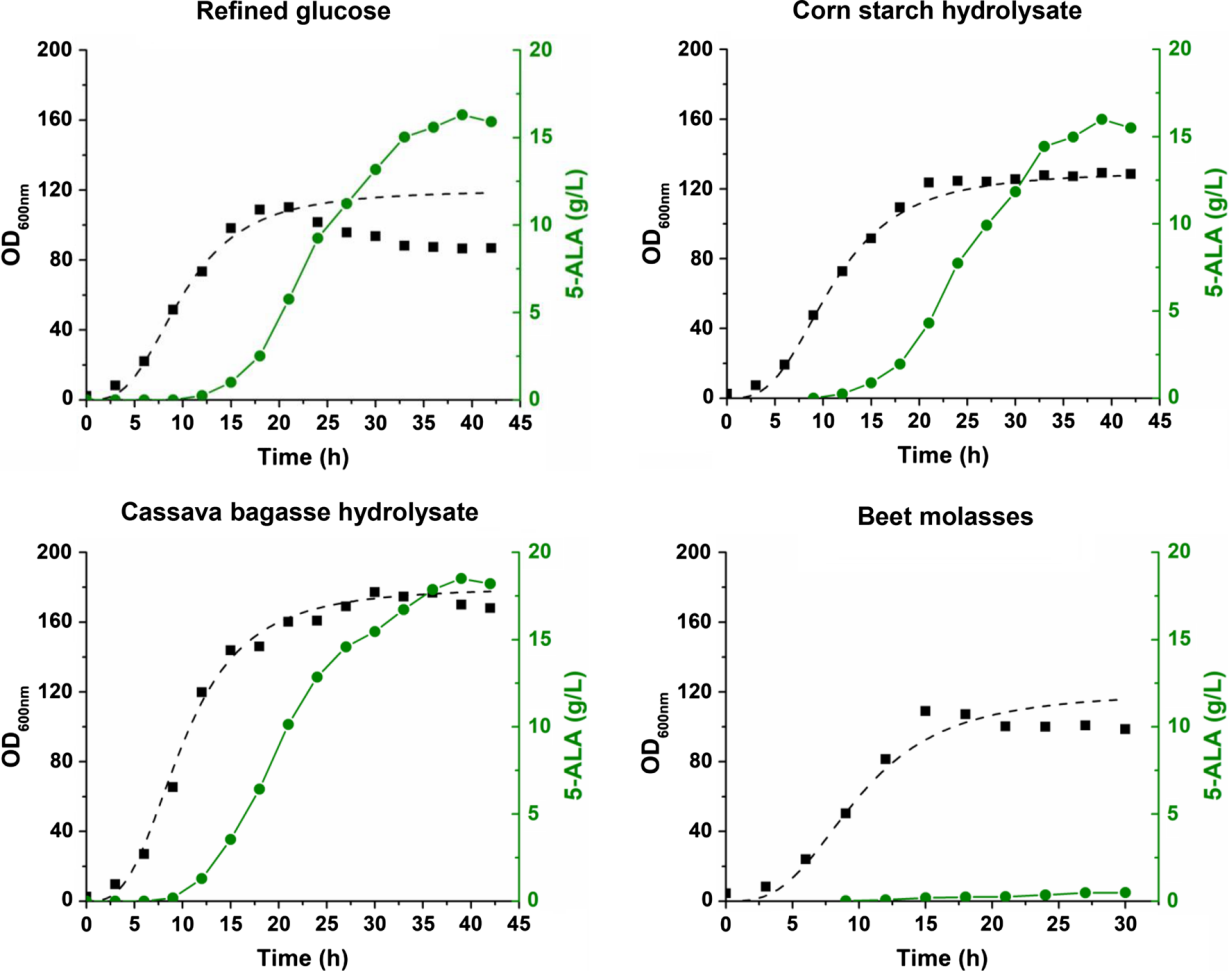
Fed-batch fermentation for 5-ALA production using different carbon sources. Black square, OD_600nm_; green circle, 5-ALA. Dotted line represents simulated growth curve based on OD_600nm_ data. Cultivation was performed in 5-L bioreactors with fermentation medium. IPTG (0.1 mM) and glycine (4 g/L) were added when OD_600nm_ reached approximately 40 to induce gene expression and 5-ALA biosynthesis. Sugar solution and glycine were continuously fed into the bioreactor during the fermentation. The pH was controlled at 6.5 initially and switched to 6.0 at 15 h. The dissolved O_2_ was maintained at 30% initially and switched to 10% at 18 h

Carbon sources often constitute a large portion of production cost in biological fermentation. Up to now, all the efforts to biologically produce 5-ALA use refined glucose as the main carbon source supplemented with a small amount of 5-ALA precursors such as succinate and glycine (Table 1). To reduce the cost of carbon source, it is desirable to explore alternative low-cost feedstocks with wide availability, such as agricultural residues and industrial wastes. We first selected an easy-to-handle feedstock with low impurity, corn starch, to replace refined glucose. α-Amylase and glucoamylase were used for liquefaction and saccharification of corn starch prior to fermentation. The concentrated hydrolysate was then used to provide carbon source for 5-ALA production by strain CA1P4. Very similar growth, 5-ALA production, and by-product formation were observed for fed-batch fermentation using corn starch hydrolysate and refined glucose. The final 5-ALA and lactate titers reached 16.0 g/L and 1.1 g/L in 39 h, respectively (Fig. 6 and Additional file 1: Fig. S2).

Next, cassava bagasse, an agricultural residue from the industrial processing of cassava for starch extraction, was used for 5-ALA fermentation. Cassava bagasse is generated in large quantities in many countries and usually treated as a solid waste or a low-value animal feed. However, high residue contents of carbohydrate components in cassava bagasse can be converted to fermentable sugars. Given its low-cost, availability and non-competition with food supply, cassava bagasse is an attractive feedstock for production of biofuels and biochemicals [46]. The cassava bagasse used in this study contains (w/w): 50.3% starch, 12.2% fiber (cellulose and hemicellulose), and 6.5% moisture. The carbohydrate components of the bagasse were mainly converted into sugars after enzymatic hydrolysis, with a yield of 0.53 g glucose/g cassava bagasse. Prior to fermentation, the hydrolysate was concentrated to a thick syrup containing approximately 420 g/L glucose by evaporation under vacuum. After fed-batch fermentation, 18.5 g/L 5-ALA was produced with a highest OD_600nm_ value of 177.2, which were 13.5% and 60.8% higher than those obtained by using refined glucose, respectively (Fig. 6). Lactate was the main by-product and its final titer (1.2 g/L) was similar with those produced using refined glucose and corn starch hydrolysate (Additional file 1: Fig. S2).

Finally, beet molasses, the syrupy by-product yielded after sugar crystallization from sugar beet juice, was tested for 5-ALA production. Beet molasses is rich in sucrose and contains small amounts of glucose and fructose. To hydrolyze the sucrose and remove undesired compounds in beet molasses, a pretreatment procedure with sulfuric acid was conducted as previously described [47]. Use of beet molasses as the carbon source did not affect the cell growth, whereas 5-ALA was hardly produced during the fermentation. After 30 h, the 5-ALA titer was only 0.5 g/L in the fermentation broth (Fig. 6). Instead, large amounts of lactate (7.1 g/L) and acetate (4.9 g/L) were produced (Additional file 1: Fig. S2), suggesting a redistribution of metabolic flux possibly caused by certain effectors in beet molasses. It has been reported that beet molasses usually contains heavy metals such as iron, zinc, copper, manganese, magnesium, and calcium. High concentrations of heavy metals in the medium may inhibit activities of enzymes associated with biosynthesis [48]. Although sulfuric acid treatment is expected to remove some heavy metals, previous reports showed that contents of iron, zinc, copper, and nickel are not reduced by the pretreatment [49]. Notably, ALAS is sensitive to most of metal ions, such as iron, zinc, copper, plumbum, and cobalt [50]. Therefore, special pretreatment is required for removing heavy metals from beet molasses before it can be used for 5-ALA production.

Among the three tested cheap bioresources, cassava bagasse is obviously the most promising feedstock for 5-ALA production due to its beneficial effects on cell growth and 5-ALA formation. This result can be explained by the presence of some nutrients like vitamins and minerals in the hydrolysate [51]. The engineered strain CA1P4 with the best fermentation performance produced 18.5 g/L 5-ALA using cassava bagasse hydrolysate. This titer is 1.6-fold higher than the previous record (11.5 g/L) obtained by engineered *E. coli* using glucose as the main carbon source in a one-step fermentation process [32] (Table 1). Furthermore, this titer is even higher (1.3-fold) than that obtained in a two-step fermentation process (14.7 g/L) [27]. Based on the fed-batch fermentation data, 9.4 kg glucose or 21.9 kg cassava bagasse would be cost for producing 1.0 kg 5-ALA. Considering the price of cassava bagasse (~ $20/ton) is much lower than that of glucose (~ $521/ton), using cassava bagasse to replace glucose will reduce the cost of carbon source by 90.1%.

## Conclusions

In this study, we showed that 5-ALA production could be greatly enhanced by accurately regulating the expression of key enzymes involved in 5-ALA biosynthetic pathway and anaplerotic pathway in *C. glutamicum*. The best producer strain CA1P4 produced the highest 5-ALA titer up to 18.5 g/L using cheap renewable bioresources including cassava bagasse in 39 h fermentation, which is the highest titer for 5-ALA bioproduction reported to date. The results bring a splendid expectation for large-scale bioproduction of 5-ALA. The metabolic engineering strategy presented here can be also applied to improve the bioproduction of other chemicals derived from TCA cycle.

## Methods

### Bacterial strains and cultivation conditions

The bacterial strains used in this study are listed in Table 2. *E. coli* DH5α was used as the host for plasmid construction and was cultivated aerobically at 37 ℃ using Luria–Bertani (LB) medium. *C. glutamicum* ATCC 13032 and its derivatives were used for 5-ALA production and were cultivated aerobically at 30 ℃ in LB medium supplement with glucose. Kanamycin at a final concentration of 25 μg/mL or 50 μg/mL was added for *C. glutamicum* or *E. coli* as required, respectively.

**Table 2:** Strains and plasmids used in this study

Strain or plasmid	Description^a^	Reference or source
Strain		
*E. coli* DH5α	F^−^*supE*44 ∆*lacU*169 (φ80 *lacZ*∆M15) *hsdR*17 *recA*1 *endA*1 *gyrA*96 *thi*-1 *relA*1	Invitrogen
*C. glutamicum* ATCC 13032	Wild type	ATCC
CA	*C. glutamicum* ATCC 13032 derivative harboring plasmid pRpA	This study
CO	*C. glutamicum* ATCC 13032 derivative harboring plasmid pRpO	This study
CA-Rs	*C. glutamicum* ATCC 13032 derivative harboring plasmid pRsA	This study
CA1	*C. glutamicum* ATCC 13032 derivative harboring plasmid pRpA1	This study
CA2	*C. glutamicum* ATCC 13032 derivative harboring plasmid pRpA2	This study
CA3	*C. glutamicum* ATCC 13032 derivative harboring plasmid pRpA3	This study
CA4	*C. glutamicum* ATCC 13032 derivative harboring plasmid pRpA4	This study
CA1P	*C. glutamicum* ATCC 13032 derivative harboring plasmid pRpA1P	This study
CA1P1	*C. glutamicum* ATCC 13032 derivative harboring plasmid pRpA1P1	This study
CA1P2	*C. glutamicum* ATCC 13032 derivative harboring plasmid pRpA1P2	This study
CA1P3	*C. glutamicum* ATCC 13032 derivative harboring plasmid pRpA1P3	This study
CA1P4	*C. glutamicum* ATCC 13032 derivative harboring plasmid pRpA1P4	This study
CA1P5	*C. glutamicum* ATCC 13032 derivative harboring plasmid pRpA1P5	This study
CA1P6	*C. glutamicum* ATCC 13032 derivative harboring plasmid pRpA1P6	This study
Plasmid		
pET28a-RS-hemA	pET28a harboring *hemA* from *R. sphaeroides* (RsHemA), Kan^R^	[52]
pEC-XK99E	*E. coli–C. glutamicum* shuttle vector, *P*_*trc*_ promoter, Km^R^	[58]
pRpA	pEC-XK99E harboring *hemA* from *R. palustris* ATCC 17001 (RpHemA)	This study
pRpO	pEC-XK99E harboring *hemO* from *R. palustris* ATCC 17001 (RpHemO)	This study
pRsA	pEC-XK99E harboring *hemA* from *R. sphaeroides* (RsHemA)	This study
pRpAtuf	pRpA derivative, *P*_*trc*_ promoter of RpHemA replaced with *P*_*tuf*_ promoter of *C. glutamicum* ATCC 13032	This study
pRpAsod	pRpA derivative, *P*_*trc*_ promoter of RpHemA replaced with *P*_*sod*_ of *C. glutamicum* ATCC 13032	This study
pRpA1	pRpA derivative, RBS-1 of RpHemA replaced with RBS-2	This study
pRpA2	pRpA derivative, RBS-1 of RpHemA replaced with RBS-3	This study
pRpA3	pRpA derivative, RBS-1 of RpHemA replaced with RBS-4	This study
pRpA4	pRpA derivative, RBS-1 of RpHemA replaced with RBS-5	This study
pRpA1P	pRpA1 derivative, harboring *ppc* from *C. glutamicum* ATCC 13032	This study
pRpA1P1	pRpA1P derivative, RBS-1 of *ppc* replaced with RBS-2	This study
pRpA1P2	pRpA1P derivative, RBS-1 of *ppc* replaced with RBS-6	This study
pRpA1P3	pRpA1P derivative, RBS-1 of *ppc* replaced with RBS-7	This study
pRpA1P4	pRpA1P derivative, RBS-1 of *ppc* replaced with RBS-8	This study
pRpA1P5	pRpA1P derivative, RBS-1 of *ppc* replaced with RBS-5	This study
pRpA1P6	pRpA1P derivative, RBS-1 of *ppc* replaced with RBS-9	This study

^a^Kan^R^ represents resistance to kanamycin. RBSs used in this study are shown in Additional file 1: Table S2

For 5-ALA fermentation in shake flasks, the recombinant *C. glutamicum* strains were inoculated into 50 mL LB medium in 500-mL shake flasks containing 20 g/L glucose and incubated at 30 ℃ and with shaking at 220 rpm for 12 h. The preculture was then inoculated into 50 mL modified M9 medium in 500 mL shake flasks to an initial OD_600nm_ of 0.5. Fermentation was performed at 30 ℃ and with shaking at 220 rpm, and the pH was maintained at 6.4–7.0 with 25% ammonium hydroxide throughout the fermentation. IPTG was added to a final concentration of 0.1 mM at 3 h to induce gene expression. The modified M9 medium contains 50 g/L glucose, 17.1 g/L Na_2_HPO_4_·12H_2_O, 6 g/L glycine, 3 g/L KH_2_PO_4_, 2 g/L yeast extract, 1 g/L NH_4_Cl, 0.5 g/L NaCl, 0.24 g/L MgSO_4_·7H_2_O, and 11 mg/L CaCl_2_.

For fed-batch fermentation in 5-L bioreactors, a 200-mL seed culture cultivated using the seed medium for 12 h was transferred into a 5-L bioreactor containing 2 L fermentation medium. Fermentation was conducted at 30 ℃ and with 30% dissolved oxygen (adjusted with aeration and agitation). After 18 h fermentation, the dissolved oxygen was maintained at 10%. Initially, ammonium hydroxide was added automatically to maintain pH at 6.5, and after 15 h fermentation the pH was maintained at 6.0. Glycine (final concentration of 4 g/L) and IPTG (final concentration of 0.1 mM) were added to induce gene expression and 5-ALA production when the OD_600nm_ reached approximately 40. Sugar syrup containing 420 g/L glucose or total sugar and 200 g/L glycine solution were fed into the bioreactor to maintain the sugar and glycine concentrations at about 10 g/L and 6 g/L, respectively. The seed medium contains 50 g/L glucose, 5 g/L yeast extract, 5 g/L urea, 3 g/L Na_2_HPO_4_·12H_2_O, 3 g/L KH_2_PO_4_, 1 g/L NH_4_Cl, 1 g/L MgSO_4_·7H_2_O, 0.5 g/L NaCl, 20 mg/L MnSO_4_·5H_2_O, 11 mg/L·CaCl_2_, 5 mg/L thiamine hydrochloride, and 0.1 mg/L biotin. The fermentation medium contains 50 g/L initial sugar, 6 g/L K_2_HPO_4_·3H_2_O, 5 g/L NH_4_Cl, 2 g/L yeast extract, 1.5 g/L KH_2_PO_4_, 1 g/L MgSO_4_·7H_2_O, 0.5 g/L NaCl, 20 mg/L MnSO_4_·5H_2_O, 11 mg/L CaCl_2_, 5 mg/L thiamine hydrochloride, and 0.1 mg/L biotin.

### Gene expression in *C. glutamicum*

The *E. coli*–*C. glutamicum* shuttle vector pEC-XK99E was used for ALAS and PPC expression in *C. glutamicum*. All the recombinant plasmids constructed and used in this study are listed in Table 2. Plasmids pRpA, pRpO, and pRsA were constructed by amplifying RpHemA and RpHemO encoding genes from *R. palustris* genomic DNA and RsHemA encoding gene from plasmid pET28a-RS-hemA [52] using primers listed in Additional file 1: Table S1. The PCR products were then digested with *Eco*RI and *Sma*I and ligated with pEC-XK99E digested with the same endonucleases. For constitutive expression of RpHemA, constitutive promoters *P*_*tuf*_ and *P*_*sod*_ were amplified and cloned into pRpA, resulting in plasmids pRpAsod and pRpAtuf, respectively. In order to optimize the expression of ALAS, the original RBS1 of ALAS gene in pRpA was replaced with four RBSs (see Additional file 1: Table S2) chosen from previously constructed libraries [38] by PCR, resulting in plasmids pRpA1-pRpA4. To overexpress PPC, *ppc* gene with RBS1 was amplified from *C. glutamicum* ATCC 13032 genomic DNA and cloned into the *Sma*I and *Xba*I sites of plasmid pRpA1, resulting in plasmid pRpA1P. To regulate PPC expression, the original RBS1 of *ppc* in plasmid pRpA1P was replaced with six RBSs (see Additional file 1: Table S2), resulting in pRpA1P1-pRpA1P6. The recombinant plasmids were transformed into *C. glutamicum* via electro-transformation. In the case of using *P*_*trc*_ to control gene expression, IPTG was added to a final concentration of 0.1 mM.

### Pretreatment of cheap bioresources

Cassava bagasse, purchased from Qingdao Aisipurui Co., Ltd (Shandong, China), was dried and milled to a fine powder with a size about 50–100 μm. The cassava bagasse hydrolysate was prepared as described previously [53]. Briefly, 10% (w/v) cassava bagasse mash was liquefied at 90 ℃ for 2 h using thermostable α-amylase (Aladdin, Shanghai, China) with a dosage of 0.1% (w/w). Then, the liquefied mash that cooled to 60 ℃ was saccharified for 2 h using glucoamylase (Aladdin, Shanghai, China) with a dosage of 0.3% (w/w). To hydrolyze the remaining cellulose, cellulase (Macklin, Shanghai, China) was added into the mixture at 0.4% (w/w) for another 12 h treatment. After hydrolysis, the mixture was centrifuged at 7000*g* for 15 min to remove the insoluble substances. The supernatant (cassava bagasse hydrolysate) was further concentrated by vacuum-rotary evaporation.

Corn starch was purchased from Shandong Xiwang Sugar Industry Co., Ltd. (Shandong, China) and its hydrolysate was prepared using the same liquefaction and saccharification processes with α-amylase and glucoamylase as hydrolysis of cassava bagasse. After hydrolysis, the mixture was centrifuged at 7000*g* for 15 min to remove the insoluble substances and concentrated by vacuum-rotary evaporation.

The beet molasses was provided by Neimenggu Fufeng Biotechnologies Co., Ltd (Neimenggu, China) and contained 53% (w/w) sugars. The molasses was diluted with water at a 3:1 ratio to prepare a molasses solution. The acid treatment process entailed the addition of sulfuric acid to adjust the pH to 2.0 and subsequent incubation in a thermal bath at 100 ℃ for 30 min. After incubation and cooling, the molasses solution was adjusted to pH 7.0 using 1 M NaOH solution. Then the molasses solution was centrifuged at 7000*g* for 15 min and the supernatant was used for fermentation [47].

### Enzyme activity assays

Cells were collected and washed with 100 mM Tris–HCl buffer (pH 7.5). Mechanical lysis of cells was performed with glass beads using FastPrep^®^‐24 Classic Instrument (MP Biomedicals, USA). The crude extract was centrifuged at 12,000*g* for 10 min at 4 ℃, and the supernatant was used for enzyme activity assay. The protein concentration was determined with BCA Protein Assay Kit (Thermo Fisher Scientific, USA). ALAS activity was determined by measuring 5-ALA formation (µmol/L min) [25]. The reaction mixture contained 100 mM Tris–HCl (pH 7.5), 200 mM glycine, 0.2 mM succinyl-CoA, 0.1 mM pyridoxal phosphate (PLP) and 20 µg crude extract. After proceeding at 37 ℃ for 10 min, the reaction was terminated by the addition of 10% (v/v) trichloroacetic acid. Concentration of 5-ALA in the supernatant was determined. PPC activity was analyzed by a coupled reaction with malate dehydrogenase at 30 °C as previously described [54]. The reaction mixture contained 100 mM Tris–HCl (pH 7.5), 2 mM phosphoenolpyruvate, 10 mM NaHCO_3_, 10 mM MnSO_4_, 0.1 mM NADH, 20% (v/v) glycerol, 1.6 U malate dehydrogenase, and 20 µg crude extract. The activity was assayed spectrophotometrically by monitoring the decrease in absorbance of NADH at 340 nm.

### Analytical methods

Cell biomass was determined by the optical density at 600 nm (OD_600nm_) with a UV-1800 spectrophotometer (Shimadzu, Kyoto, Japan) after proper dilution with distilled water. Glucose concentration was measured using SBA-40 (Biology Institute of Shandong Academy of Sciences, Shandong, China). The total sugar concentration was analyzed using the phenol/sulfuric acid method [55]. The concentration of 5-ALA in the fermentation broth was determined following the method described by Mauzerall and Granick [56]. Organic acids were analyzed by using HPLC equipped with a UV detector (Shimadzu, Japan) and a Bio-Rad Aminex HPX‐87H column (300 × 7.8 mm) according to the procedure described previously [57]. The 5-ALA yield was defined as (mole of 5-ALA produced/mole of glucose consumed) × 100%.

## Supplementary information

**Additional file 1: Figure S1.** Plasmid pRpA1P4 used for *hemA* and *ppc* overexpression in strain CA1P4. **Figure S2.** By-product lactate and acetate of fed-batch fermentations using different carbon sources. **Table S1.** Primers used in this study. **Table S2.** RBSs used in this study.

## Data Availability

All data generated or analyzed in this study are included in this article and its Additional information files. The datasets used and/or analyzed during the current study are available from the corresponding author on reasonable request.

## Abbreviations

5-ALA: 5-Aminolevulinic acid
ALAS: 5-ALA synthetase
IPTG: Isopropyl-β-d-thiogalactopyranoside
RBS: Ribosome binding site
PLP: Pyridoxal phosphate
PPC: Phosphoenolpyruvate carboxylase
