# Supplementary material for: Efficient bioproduction of 5-aminolevulinic acid, a promising biostimulant and nutrient, from renewable bioresources by engineered Corynebacterium glutamicum
Source: Biotechnol Biofuels. 2020 Mar 10;13:41. doi: 10.1186/s13068-020-01685-0 (PMC7063817; doi:10.1186/s13068-020-01685-0)
Supplement: Supplementary file 1 — Additional file 1: Figure S1. Plasmid pRpA1P4 used for hemA and ppc overexpression in strain CA1P4. Figure S2. By-product lactate and acetate of fed-batch fermentations using different carbon sources. Table S1. Primers used in this study. Table S2. RBSs used in this study. [file 13068_2020_1685_MOESM1_ESM.doc]

**Additional file 1**

**
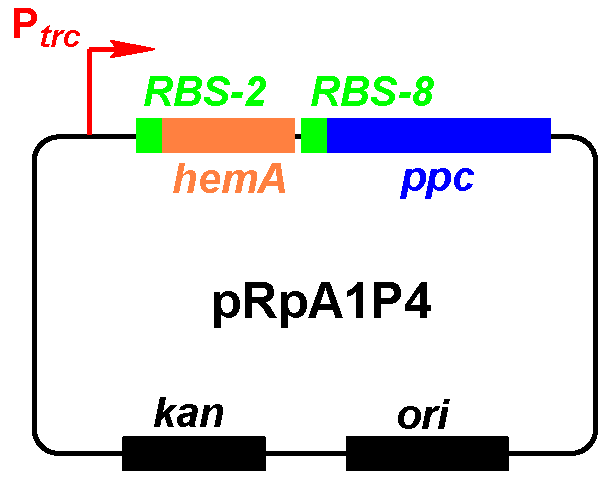
**

**Fig. S1** Plasmid pRpA1P4 used for *hemA* and *ppc* overexpression in strain CA1P4. P*_trc_*, IPTG-inducible *trc* promoter; *hemA*, 5-aminolevulinate synthase (ALAS) encoding gene; *ppc*, phosphoenolpyruvate carboxylase (PPC) encoding gene; RBS-2, AAAGGAGGTTGTC; RBS-8, AAAGGCTGGAATT.

**
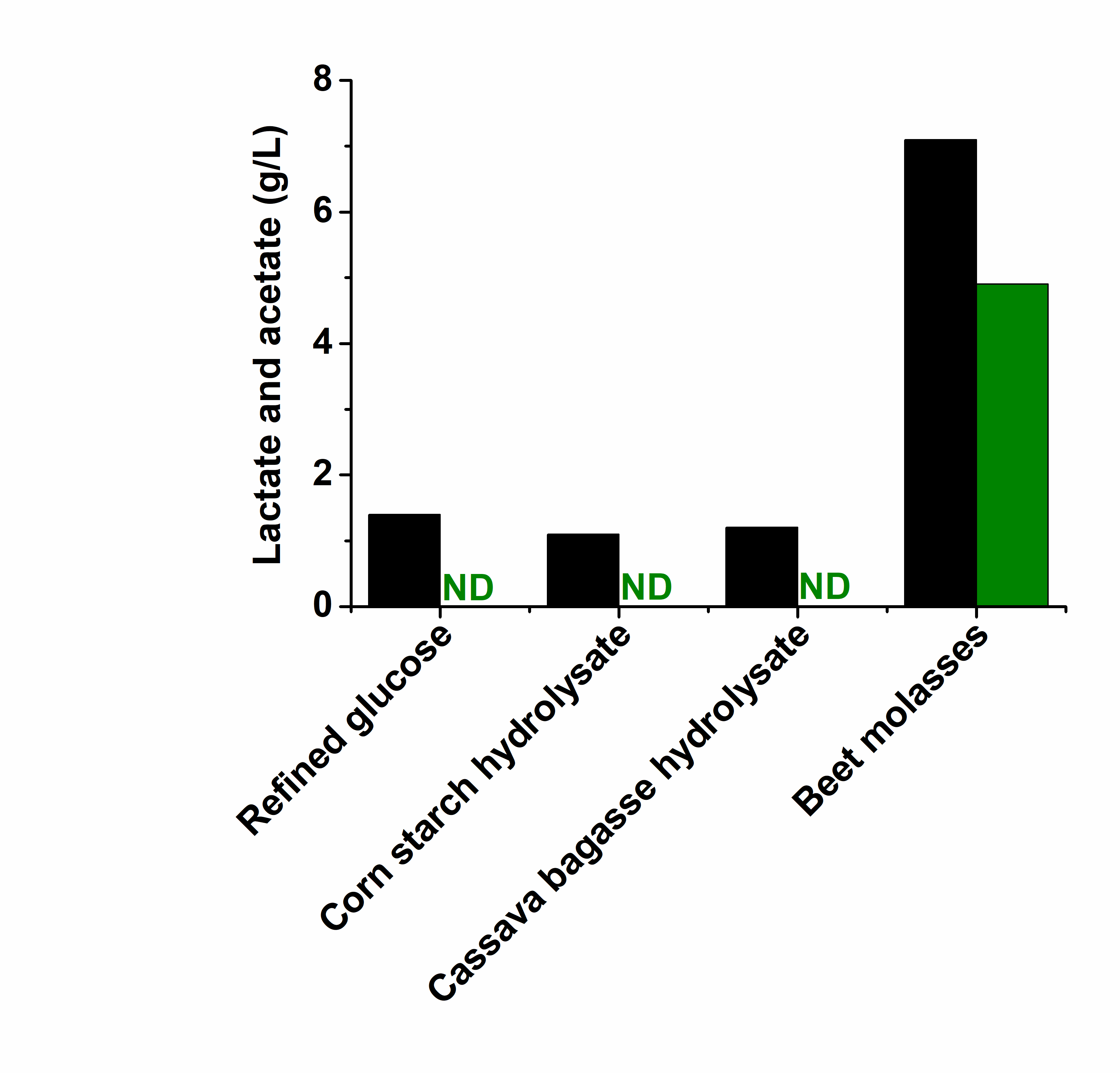
**

**Fig. S2** By-products lactate and acetate of fed-batch fermentations using different carbon sources. Lactate and acetate concentrations were determined at the end of fed-batch fermentations (Fig. 6) using HPLC. Black bars, lactate; green bars, acetate. ND represents acetate is not detected in the fermentation broth.

**Table S1** Primers used in this study

| **Primer** | **Sequence (5’-3’)^a^** | **Relevance** |
| --- | --- | --- |
| *RphemA*-F | GGCGAATTC*AAGGAGATATAGAT***ATG**AATTACGAAGCCTATTTCCGCCGT | pRpA |
| *RphemA*-R | TATACCCCGGGTCAGGCCGCCTTGGCGAGAC |
| *RphemO*-F | GGCGAATTC*AAGGAGATATAGAT***ATG**CAATACAACAAATTCTTCGAAGAC | pRpO |
| *RphemO*-R | TATACCCCGGGCTACTCCGCCGCGATCGC |
| *RshemA*-F | CGAGCTC*AAGGAGATATAGAT***ATG**GACTACAACCTGGCGCTG | pRsA |
| *RshemA*-R | CGCGGATCCGAATTCCGCAACAACTTCCG |
| P*tuf-F* | GCTCATCCATATGTGGCCGTTACCCTGCGAATG | pRpAtuf |
| P*tuf-R* | CCGGAATTCTGTATGTCCTCCTGGACTTC |
| P*sod-F* | GCGGTTCCATATGTAGCTGCCAATTATTCCGGG | pRpAsod |
| P*sod-R* | CGCGAATTCTGGGTAAAAAATCCTTTCG |
| RBS2-*RphemA*-F | GGCGAATTC*AAAGGAGGTTGTC***ATG**AATTACGAAGCCTATTTCCGCCGT | pRpA1 |
| RBS3-*RphemA*-F | GGCGAATTC*AAAGGAGCGGTCC***ATG**AATTACGAAGCCTATTTCCGCCGT | pRpA2 |
| RBS4-*RphemA*-F | GGCGAATTC*AAAGGAGGATTAG***ATG**AATTACGAAGCCTATTTCCGCCGT | pRpA3 |
| RBS5-*RphemA*-F | GGCGAATTC*AAAGGAGTTGCTT***ATG**AATTACGAAGCCTATTTCCGCCGT | pRpA4 |
| RBS1-*ppc*-F | TCCCCCGGGGTCGAC*AAGGAGATATAGAT***ATG**ACTGATTTTTTACGCGATGAC | pRpA1P |
| *ppc*-R | CTAGTCTAGACTAGCCGGAGTTGCGCAGCGCAGT |
| RBS2-*ppc*-F | TCCCCCGGGGTCGAC*AAAGGAGGTTGTC***ATG**ACTGATTTTTTACGCGATGAC | pRpA1P1 |
| RBS6-*ppc*-F | TCCCCCGGGGTCGAC*AAAGGAATTGGC***ATG**ACTGATTTTTTACGCGATGAC | pRpA1P2 |
| RBS7-*ppc*-F | TCCCCCGGGGTCGAC*AAAGGTTTCAAGT***ATG**ACTGATTTTTTACGCGATGAC | pRpA1P3 |
| RBS8-*ppc*-F | TCCCCCGGGGTCGAC*AAAGGCTGGAATT***ATG**ACTGATTTTTTACGCGATGAC | pRpA1P4 |
| RBS5-*ppc*-F | TCCCCCGGGGTCGAC*AAAGGAGTTGCTT***ATG**ACTGATTTTTTACGCGATGAC | pRpA1P5 |
| RBS9-*ppc*-F | TCCCCCGGGGTCGAC*AAAGGGTATTGGC***ATG**ACTGATTTTTTACGCGATGAC | pRpA1P6 |

^a^Underlined, italic, and bold letters represent restriction site, RBS sequence, and start codon, respectively.

**Table S2 RBSs used in this study**

| **RBS** | **Sequence (5’-3’)** |
| --- | --- |
| RBS-1 | AAGGAGATATAGAT |
| RBS-2 | AAAGGAGGTTGTC |
| RBS-3 | AAAGGAGCGGTCC |
| RBS-4 | AAAGGAGGATTAG |
| RBS-5 | AAAGGAGTTGCTT |
| RBS-6 | AAAGGAATTGGC |
| RBS-7 | AAAGGTTTCAAGT |
| RBS-8 | AAAGGCTGGAATT |
| RBS-9 | AAAGGGTATTGGC |
